# Primary Brain Calcification Causal PiT2 Transport-Knockout Variants can Exert Dominant Negative Effects on Wild-Type PiT2 Transport Function in Mammalian Cells

**DOI:** 10.1007/s12031-016-0868-7

**Published:** 2016-12-09

**Authors:** Frederik Tibert Larsen, Nina Jensen, Jacob Kwasi Autzen, Iben Boutrup Kongsfelt, Lene Pedersen

**Affiliations:** 1 Department of Molecular Biology and Genetics, Aarhus University, C. F. Møllers Allé 3, Building 1130, 8000 Aarhus, Denmark; 2 Department of Clinical Medicine, Aarhus University, Aarhus, Denmark

**Keywords:** *SLC20A2*, Primary familial brain calcification, Phosphate transporter, PiT2, Oligomerization

## Abstract

Primary brain calcification (PBC) is a neurodegenerative disorder characterized by calcium-phosphate deposits in the basal ganglia and often also other areas of the brain. The prevalent clinical manifestations are cognitive impairment, neuropsychiatric symptoms, and movement disorders. In recent years, monoallelic variants in *SLC20A2*, which encodes the type III sodium-dependent inorganic phosphate (P_i_) transporter 2 (PiT2), have been linked to the familial form of PBC in 40–50% of the families reported worldwide as well as to sporadic cases of PBC. Further insight into the disease mechanism is, however, needed. Based on co-expression studies of wild-type and variant PiT2 in *Xenopus laevis* oocytes, the molecular disease mechanism associated with *SLC20A2* missense variants has formerly been suggested to be haploinsufficiency. We have here used mammalian cells isolated from a *Slc20a2* ^*−/−*^ mouse and co-expression of human wild-type and variant PiT2. Two of the variants studied have both been reported twice in unrelated PBC cases: PiT2D28N in two sporadic cases and PiT2E575K in a familial and a sporadic case. We find that in mammalian cells, the analyzed *SLC20A2* missense variants can exert their effect in a dominant negative manner resulting in decreased wild-type PiT2 P_i_ transport. Thus, compared to monoallelic lack of functional PiT2 protein expression, which reasonably points towards haploinsufficiency, certain *SLC20A2* missense variants may be more detrimental for cellular P_i_ uptake and potentially contribute to an earlier disease onset and/or a more severe phenotype as observed for *Slc20a2* ^*−/−*^ mice compared to *Slc20a2* ^*+/−*^ mice.

## Introduction

Primary familial brain calcification (PFBC) (OMIM: #213600, #616413, #615007, and #615483), formerly known as “Fahr’s disease” or familial idiopathic basal ganglia calcification (FIBGC), is a rare autosomal dominant inherited neurodegenerative disease. It is characterized by bilateral calcifications in the basal ganglia, but other areas of the brain are also often affected. Primary refers to that the calcifications are not secondary to systemic diseases, infections, traumas, or toxicity (Sobrido et al. 2014; Westenberger and Klein 2014). The symptoms associated with PFBC are heterogeneous with the most prevalent being cognitive impairment (ranging from mild cognitive impairment to dementia), neuropsychiatric symptoms (e.g., mood disorders and psychotic signs), and movement disorders (often parkinsonism), but other symptoms as migraine and speech disorders can also be present (Manyam et al. 2001; Nicolas et al. 2013a; Sobrido et al. 2014; Yamada et al. 2014; Nicolas et al. 2015). The age of onset of clinical symptoms is also diverse and ranges from early childhood to old age (Nicolas et al. 2013a; Sobrido et al. 2014; Nicolas et al. 2015).

The gene *SLC20A2* encodes a member of the inorganic phosphate (P_i_) transport (PiT) family (TC#2.A.20), the type III sodium-dependent P_i_ transporter 2 (PiT2) (Kavanaugh et al. 1994; Kavanaugh and Kabat 1996; Bai et al. 2000). PiT2 is ubiquitously expressed in mammalian cells and assigned a role in cellular P_i_ homeostasis and, recently, in maintaining the lower P_i_ concentration in the cerebrospinal fluid (CSF) compared to the blood (Kavanaugh et al. 1994; Uckert et al. 1998; Guerreiro et al. 2014; Jensen et al. 2016). In 2012, Wang et al. linked a deletion and five missense variants in *SLC20A2,* all resulting in PiT2 proteins with impaired P_i_-transport function, as well as a *SLC20A2* frameshift variant to PFBC (Wang et al. 2012). It was later confirmed that knockout (KO) of PiT2 in mice indeed led to brain calcifications, which could be found associated with the vasculature as observed in autopsied PFBC patients (Miklossy et al. 2005; Wider et al. 2009; Jensen et al. 2013). Variants in three other genes have also been associated with PFBC: the genes encoding the platelet-derived growth factor receptor β (PDGF-Rβ) and its main ligand, PDGF-B (Keller et al. 2013; Nicolas et al. 2013b), and lately, the gene encoding the P_i_ exporter, XPR1 (Legati et al. 2015). *SLC20A2* variants are present in 40 to 50% of the PFBC families (Hsu et al. 2013; Yamada et al. 2014), which makes variants in this gene a prime cause of PFBC. In addition, *SLC20A2* variants are also linked to sporadic cases of primary brain calcification (PBC), confirmed or not confirmed as de novo variants (Schottlaender et al. 2012; Chen et al. 2013; Nicolas et al. 2013a; Ferreira et al. 2014; Westenberger and Klein 2014; Lemos et al. 2015).

Human PiT2 (NP_006740.1) is a 652-amino acid long protein (van Zeijl et al. 1994). The N- and C-terminal parts are multi-membrane spanning and connected with a large intracellular domain comprising approximately half the protein (van Zeijl et al. 1994). Since 2012, various kinds of variants in *SLC20A2* have been reported linked to familial and sporadic PBCs, e.g., missense, frameshift, deletions, nonsense, and splice site (Schottlaender et al. 2012; Wang et al. 2012; Chen et al. 2013; Hsu et al. 2013; Lemos et al. 2013; Nicolas et al. 2013a; Ferreira et al. 2014; Taglia et al. 2014; Westenberger and Klein 2014; Yamada et al. 2014; Lemos et al. 2015; Nicolas et al. 2015; Liu et al. 2016). They are in general scattered over the coding sequence with the exception of exon 6, which is predicted to encode the transmembrane region positioned immediately N-terminal to the large intracellular domain (Bøttger and Pedersen 2011; Westenberger and Klein 2014; Lemos et al. 2015). More than 50% of the presently reported *SLC20A2* variant types are missense variants (Westenberger and Klein 2014; Lemos et al. 2015). Of these, the missense variants p.Asp28Asn (PiT2D28N) and p.Glu575Lys (PiT2E575K) had previously been investigated and shown not to support P_i_ uptake in *Xenopus laevis* oocytes (Bøttger and Pedersen 2002, 2005); D28 and E575 were originally selected for investigation due to their high degree of phylogenetic conservation with D28 being part of the N-terminal PiT family signature sequence (Bøttger and Pedersen 2002, 2005). In their seminal study of PFBC families reported in 2012, Wang et al. identified PiT2E575K as well as additional *SLC20A2* missense variants, which they also found not to support P_i_ uptake in *Xenopus* oocytes (Wang et al. 2012). The authors furthermore analyzed whether PiT2E575K and the missense variant p.Ser601Trp (PiT2S601W) acted in dominant negative manners on the P_i_-uptake function of wild-type (WT) PiT2 by co-injection of complementary RNAs encoding WT PiT2 and either of the two variants at a 1:1 ratio and found this not to be the case (Wang et al. 2012). Therefore, the general view of how *SLC20A2* variants can lead to disease is by haploinsufficiency (Westenberger and Klein 2014; Lemos et al. 2015). In the cases where there is no protein expression from the variant allele, haploinsufficiency is the likely disease mechanism. It has, however, been shown that PiT2 can form functional oligomers in mammalian cells and, using P_i_-transport-incompetent PiT2 proteins, that the oligomerization per se is independent of PiT2’s P_i_-transport ability (Salaün et al. 2002; Salaün et al. 2004). Moreover, kinetic analysis of human PiT2 expressed in *Xenopus* oocytes by Bøttger et al. revealed positive cooperativity (Hill coefficient of 2) (Bøttger et al. 2006), thus further suggesting that the transporting form of PiT2 is a dimer. It can, however, also be observed that the molecular weight of human PiT2 oligomers isolated from *Xenopus* oocytes seems to be lower than that of oligomers isolated from mammalian cells (Bøttger and Pedersen 2002; Salaün et al. 2002; Salaün et al. 2004; Bøttger and Pedersen 2005), which might implicate, e.g., post-translational processing in mammalian cells. There are indeed differences between *Xenopus* oocytes and mammalian cells, e.g., in membrane composition and expression levels of endogenous proteins (Wagner et al. 2000; Hill et al. 2005). Thus, albeit that P_i_-transport-incompetent PiT2 variants linked to PBC have been reported not to affect the P_i_-transport of WT PiT2 in *Xenopus* oocytes, we wished to address whether P_i_-transport-incompetent PiT2 variants can have a dominant negative effect in mammalian cells.

## Materials and Methods

### Cells

C57BL/6N^Tac^-*Slc20a2* ^tm1a(EUCOMM)Wtsi^/Ieg (EM:05549) (*Slc20a2* ^*+/−*^) mice obtained from the European Mouse Mutant Archive (EMMA) in Germany have been described previously (http://www.knockoutmouse.org/martsearch/project/24503) (Jensen et al. 2013). The mice were handled according to the Danish law on animal experimentation and the animal welfare policy at Aarhus University. The strain is heterozygous for the KO cassette, L1L2-PGK-P, which introduces splice acceptor and SV40 polyadenylation sequences in *Slc20a2* between the third and fourth exons (i.e., the second and third coding exons). Skin fibroblasts (3463T3) were obtained from a mouse homozygous for the KO cassette (*Slc20a* ^*−/−*^ mice); further characterization of the *Slc20a* ^*−/−*^ cells will be published elsewhere. The cells were cultivated in Dulbecco’s modified eagle’s medium (DMEM) with pyruvate (Gibco, Life Technologies) supplemented with 15% fetal bovine serum (Gibco) and 1% penicillin and streptomycin (Gibco).

### Transfection

The eukaryotic expression vector pcDNA1A^R^tkpA encoding WT human PiT2 (pOJ74) or P_i_-transport-KO mutants (pOJ74D28N, pOJ74H502A, and pOJ74E575K) have been described (Pedersen et al. 1995; Bøttger and Pedersen 2002, 2005, 2011). Cells were seeded in four-well plates (NUNC, Hounisen) at a density of 1.5 × 10^4^ cells/cm^2^ and incubated for 24 h before they were transfected using the Lipofectamine® 2000 reagent (Invitrogen™, Thermo Fisher Scientific) at a DNA/transfection reagent ratio of 1:3. This optimal ratio for transfection was determined by transfecting the cells with a eukaryotic expression vector encoding enhanced green fluorescent protein (pEGFP-N1 (Clontech)). DNAs of similar quality of pOJ74 and pOJ74D28N, pOJ74H502A, or pOJ74E575K were mixed in equimolar or different ratios. In order to avoid promotor competition, empty vector DNA, pcDNA1A^R^tkpA, was added to obtain equimolar quantities of CMV-promotor-containing constructs. In addition, pUC19 DNA was added to obtain a final concentration of 2.5 μg of DNA per well. Mock-transfected cells were transfected with empty vector DNA, pcDNA1A^R^tkpA. The transfections were performed according to the manufacturer’s instructions.

### Phosphate Uptake

Cells seeded in four-well plates and transfected as described above were incubated for 48 h after the transfection. The cells were washed in 37 °C pre-warmed P_i_-free DMEM medium (Gibco) and thereafter incubated at room temperature for 5 min in 37 °C pre-warmed P_i_-free DMEM supplemented with KH_2_^32^PO_4_ (NEX060, 1 Ci/mmol, Perkin Elmer) and cold P_i_ added to a final concentration of 1 mM P_i_. They were then washed in 3× ice-cold 0.9% NaCl and lysed with 0.5% Triton X-100. One third of each lysate was transferred to counting vials, mixed with 5 mL liquid scintillation cocktail (Optiphase Hisafe 3 (Perkin Elmer)), and counted in a liquid scintillation counter. Dilution series of ^32^P_i_ were counted simultaneously. The remaining lysates were stored at −20 °C for later protein determination using the BCA™ Protein Assay Kit (Pierce).

### Statistics

**Fig. 1:**
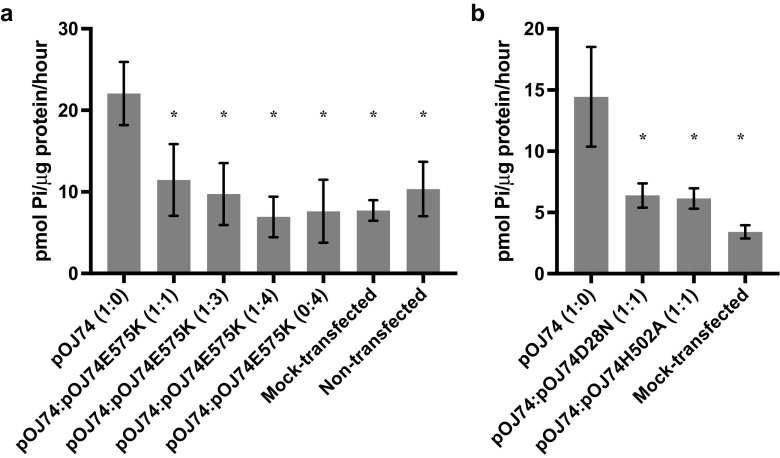
Co-expression assays. Cells were seeded at 1.5 × 10^4^ cells/cm^2^ in four-well plates. The following day, they were transfected with WT PiT2-encoding vector, pOJ74, and vectors encoding the P_i_-transport-incompetent **a** PiT2E575K (pOJ74E575K) or **b** PiT2D28N (pOJ74D28N) or PiT2H502A (pOJ74H502A) as indicated. Mock-transfected: cells transfected with empty vector DNA. Ratios refer to the ratio between vectors encoding WT and P_i_-transport-incompetent PiT2, where 1 represents 0.05 μg DNA. Emtpy expression vector and plasmid DNAs were included to ensure equimolar amounts of expression vector and same amount of DNA, respectively, in the transfections. Approximately 48 h after transfection, the cells were incubated in transport medium containing a total [P_i_] of 1 mM and the import of ^32^P_i_ was analyzed over 5 min. Results are shown as mean P_i_ uptake per microgram protein per hour of at least three wells; error bars represent SDs. Asterisk indicates *p* ≤ 0.05 relative to cells expressing WT PiT2 only (pOJ74 (1:0) (*leftmost column*))

Data are presented as mean ± standard deviation (SD). As these types of experiments can be prone to large variations, the modified Thompson tau technique was applied; one outlier in the experiment shown in Fig. 1b was discarded (*p* ≤ 0.01). The hypothesis that two mean values were identical was tested by a two-tailed Student’s *t* test; a *p* value ≤0.05 was considered statistically significant. Bonferroni-Holm correction was performed to avoid α-error accumulation (Holm 1979).

## Results

In order to address whether a P_i_-transport-incompetent PiT2 variant can affect the P_i_-transport function of the WT PiT2 protein in mammalian cells, we used PiT2E575K, which has been reported in a Spanish family as well as in a sporadic case and experimentally verified to be P_i_-transport incompetent (Bøttger and Pedersen 2002; Schottlaender et al. 2012; Wang et al. 2012). To simulate the effect of a monoallelic variant in the *SLC20A2* gene and thus mimic the PBC disease situation, we used cells (3463T3) isolated from a *Slc20a* ^*−/−*^ mouse and co-transfection of vectors expressing variant and WT human PiT2 proteins; the 3463T3 cells do not express endogenous PiT2 in the plasma membrane (Jensen et al. submitted manuscript). Transient transfection of the expression vector encoding WT PiT2 (pOJ74) was sufficient to obtain an increased P_i_-uptake ability of 3463T3 cultures (compare pOJ74 and mock- and non-transfected in Fig. 1). Co-transfection of the WT PiT2-encoding vector (pOJ74) with equimolar amounts (1:1) or three (1:3) or four (1:4) times more of the PiT2E575K-encoding vector (pOJ74E575K), however, resulted in significant decreases in the P_i_-uptake ability of the cultures compared to cultures transfected with only WT PiT2-encoding vector (pOJ74) (Fig. 1a). To analyze whether the presence of PiT2 proteins with other missense variants can mitigate the WT PiT2-mediated P_i_-uptake ability when expressed in a mammalian cell, PiT2D28N and PiT2H502A were employed. The D28N variant has been reported in two sporadic cases of PBC (Chen et al. 2013; Nicolas et al. 2013a), and the H502A mutation was employed to represent a reported variant of H502 in PFBC (H502Q in PFBC) (Hsu et al. 2013). Both PiT2D28N and PiT2H502A have been shown to be unable to support P_i_ uptake in *Xenopus* oocytes (Bøttger and Pedersen 2005, 2011). Like E575 and D28, H502 also shows a high degree of phylogenetic conservation, and it is part of the C-terminal PiT family signature sequence. Co-transfection of equimolar amounts (1:1) of the PiT2D28N- or PiT2H502A-encoding vectors (pOJ74D28N and pOJ74H502A, respectively) with the WT PiT2-encoding vector (pOJ74) also resulted in significant decreases in the P_i_-uptake ability of the cultures compared to cultures transfected with only WT PiT2-encoding vector (pOJ74) (Fig. 1b). Hence, these results show that the presence of a P_i_-transport-incompetent PiT2 protein can exert a dominant negative effect on WT PiT2-mediated P_i_-uptake in mammalian cells.

## Discussion

The here observed dominant negative effects of P_i_-transport-incompetent PiT2 proteins on WT PiT2 P_i_-transport function, when transfected in equimolar amounts, show that expression of PiT2 variants associated with familial and sporadic PBCs can exert dominant negative effects on WT PiT2 P_i_-transport function in mammalian cells. Thus, these results suggest that in association with PBC, the investigated variants can result in a more severely impaired cellular P_i_-transport function than mere lack of expression of a functional protein from one allele, i.e., haploinsufficiency.

PiT2 is also a receptor for gammaretroviruses (Kavanaugh et al. 1994; Miller and Miller 1994; van Zeijl et al. 1994), and all PiT2-derived proteins analyzed in the present study support gammaretroviral infection in mammalian cells (Bøttger and Pedersen 2002, 2005, 2011), which is interpreted as they can be inserted “correctly” in the plasma membrane. Since former characterization studies suggest that WT PiT2 proteins form functional oligomers (Salaün et al. 2002; Bøttger et al. 2006) and that oligomerization is independent of their P_i_-transport function per se (Salaün et al. 2004), we suggest that oligomerization of WT and P_i_-transport-incompetent PiT2 proteins is the underlying basis for the dominant negative effect on WT PiT2 P_i_-transport function observed in the present study.

A recent publication on an autopsied PFBC patient heterozygous for a *SLC20A2* missense variant (p.Ser637Arg) reported in two Japanese families is indeed congruent with a dominant negative effect exerted by a PiT2 variant protein (Yamada et al. 2014; Kimura et al. 2015). Kimura et al. found PiT2 expression to range from severely reduced to absent in samples from the frontal cortex, putamen, and cerebellum, i.e., the PiT2 protein amount was more than halved. Mere haploinsufficiency is expected to lead to at the most halved expression levels or less than halved due to putative compensatory upregulation of expression. The most likely explanation for the severely reduced PiT2 amount reported for the patient is that WT PiT2 protein oligomerized with the variant PiT2 protein is recognized as a misfolded confirmation by the cells and degraded.

Both the PiT2E575K and the PiT2D28N variants analyzed here have been found in two independent cases, which are likely unrelated based on their ethnic origin (Schottlaender et al. 2012; Wang et al. 2012; Chen et al. 2013; Nicolas et al. 2013a; Westenberger and Klein 2014; Lemos et al. 2015). Nevertheless, the small number of PFBC and sporadic PBC cases linked to the various *SLC20A2* variant types and the high degree of variation in onset of symptoms and their severity, even within PFBC families (Hsu et al. 2013; Nicolas et al. 2013a; Westenberger and Klein 2014; Yamada et al. 2014; Lemos et al. 2015; Nicolas et al. 2015), make studies of variant–phenotype correlations difficult. However, results from in vitro studies, as the present study, and animal studies can provide insight into potential disease mechanisms and the specific roles of PiT2. It was recently shown by Jensen et al. that the CSF of 3-week-old *Slc20a2* ^−/−^ mice on average had more than twofold elevated [P_i_] compared to WT mice (Jensen et al. 2016). Wallingford et al. have later reported similar findings in 1-year-old *Slc20a2* ^−/−^ mice, while *Slc20a* ^*+*/−^ mice were found to show an intermediate CSF [P_i_] albeit not statistically significantly different from WT mice (Wallingford et al. 2016). The elevated [P_i_] in the CSF is ascribed to a function of PiT2 in the choroid plexus as an exporter of P_i_ from the CSF (Guerreiro et al. 2014; Jensen et al. 2016; Wallingford et al. 2016). In PBC patients, a missense variant exerting a dominant negative effect could result in a potentially more severe outcome than haploinsufficiency with respect to elevation of the CSF [P_i_] as well as with respect to the putative direct effect on the cells of the brain. In addition, compared to *Slc20a2* ^*−/−*^ mice, *Slc20a2* ^*+/−*^ mice show significant less (Wallingford et al. 2016) or rarely (Jensen et al. submitted manuscript) brain calcification and at later ages (Jensen et al. 2013; Wallingford et al. 2016) (Jensen et al. submitted manuscript). And while there is a high degree of variation in the degree of calcification and the manifestation of symptoms even within PFBC families (Hsu et al. 2013; Nicolas et al. 2013a; Westenberger and Klein 2014; Yamada et al. 2014; Lemos et al. 2015; Nicolas et al. 2015), there is a correlation between the total calcification score and presence of symptoms (Nicolas et al. 2013a; Nicolas et al. 2015). Thus, missense variants exerting a dominant negative effect might contribute to an earlier disease onset and/or a more serious phenotype than variants, which cause monoallelic lack of expression of functional protein.

In conclusion, our results strongly indicate that the molecular mechanism of disease in familial and sporadic PBCs might not be ascribed solely to haploinsufficiency. Missense variants in *SLC20A2* linked to the disease have here been shown to exert a dominant negative effect on WT PiT2-mediated cellular P_i_ uptake. PiT2 proteins can oligomerize, and the variant proteins might exert their dominant negative effect, either by reducing the amount of P_i_-transport-capable PiT2 oligomers in the cell membrane and/or, as observed by Kimura et al. (Kimura et al. 2015) in a PFBC patient, by reducing the amount of WT and variant PiT2 proteins in the cells.
